# A Component-Resolved Therapeutic Vaccine for Cockroach Allergy Made of Per a 9 and Transforming Growth Factor-β Homologue, an Immunosuppressive Protein of *Brugia malayi*

**DOI:** 10.3389/fimmu.2021.676558

**Published:** 2021-05-31

**Authors:** Pannathee Prangtaworn, Kodchakorn Mahasongkram, Atiporn Saeung, Urai Chaisri, Watee Seesuay, Onrapak Reamtong, Anchalee Tungtrongchitr, Wanpen Chaicumpa, Nitat Sookrung

**Affiliations:** ^1^Graduate Program in Immunology, Department of Immunology, Faculty of Medicine Siriraj Hospital, Mahidol University, Bangkok, Thailand; ^2^Center of Research Excellence on Therapeutic Proteins and Antibody Engineering, Department of Parasitology, Faculty of Medicine Siriraj Hospital, Mahidol University, Bangkok, Thailand; ^3^Department of Parasitology, Faculty of Medicine, Chiang-Mai University, Chiang Mai, Thailand; ^4^Department of Tropical Pathology, Faculty of Tropical Medicine, Mahidol University, Bangkok, Thailand; ^5^Department of Tropical Molecular Biology and Genetics, Faculty of Tropical Medicine, Mahidol University, Bangkok, Thailand; ^6^Biomedical Research Incubation Unit, Department of Research, Faculty of Medicine Siriraj Hospital, Mahidol University, Bangkok, Thailand

**Keywords:** *Brugia malayi*, transforming growth factor-beta homologue, cockroach allergy, immunotherapy, *Periplaneta americana*, Per a 9, therapeutic vaccine

## Abstract

Allergen-specific-immunotherapy (ASIT) can cause long-term resolution of allergic diseases, reduces drug use and chances of new allergen sensitization. Nevertheless, therapeutic vaccine and data on ASIT efficacy for cockroach (CR) allergy are relatively scarce. In this study, efficacy and mechanism of a novel intranasal vaccine consisting of liposome (L)-entrapped mixture of American CR (*Periplaneta americana*) major allergen (Per a 9) and immunosuppressive protein of *Brugia malayi* nematode named transforming growth factor-beta homologue (TGH) in treatment of CR allergy were investigated along with two other vaccines (L-Per a 9 alone and L-TGH alone). All three vaccines could reduce pathogenic type 2 response and lung immunopathology in the vaccines-treated CR-allergic mice, but by different mechanisms. L-Per a 9 caused a deviation of the pathogenic type 2 to type 1 response (*IFN-γ-*upregulation), whereas the L-(TGH + Per a 9) and L-TGH generated regulatory immune responses including up-expression of immunosuppressive cytokine genes and increment of serum adenosine and lung indoleamine-2,3-dioxygenase-1 which are signatures of regulatory T cells (Tregs) and tolerogenic dendritic cells, respectively. The L-(TGH + Per a 9) should be further evaluated towards clinical application, as this vaccine has a propensity to induce broadly effective therapeutic effects for inhalant allergies.

## Introduction

Cockroach (CR) allergy is a worldwide health problem with increasing prevalence. Exposure to CR-allergens leads to allergy sensitization, especially among genetically prone/atopic subjects and young children (1). CR-sensitized individuals develop predominantly the type 2 immune response (2) by generating allergen-specific effector Th2 cells that secrete high levels of type 2 cytokines, e.g., IL-4, IL-5, and IL-13. These cytokines influence B cells to produce allergen-specific IgE, which binds to high-affinity FcϵRI on the mast cell surface. IgE cross-linked by allergen at re-exposure causes the mast cells to release vasoactive mediators, resulting in tissue inflammation and allergic manifestations that can be severe and even life threatening, like asthma. CR-allergic morbidity is more severe and prolonged than other inhalant allergies (3) and contributes to a high asthma prevalence (4). Asthmatic subjects suffer from chronic airway inflammation and tissue remodeling characterized by epithelial cell hyperplasia, proliferation and hypersecretion of mucus-producing goblet cells, airway wall thickening from collagen deposition, smooth muscle cell hypertrophy and degeneration, and inflammatory cell infiltration (5). Currently, different asthma endotypes have been recognized, based on the predominant types of infiltrated inflammatory cells into the inflamed airway (6–8) including eosinophilic/atopic asthma, neutrophilic/non-atopic asthma, mixed granulocytic asthma, paucigranulocytic asthma, type 2-high/low asthma, and Th2/Th17-predominant/neutrophilic-Th2/Th17-low asthma. Different asthma phenotypes respond differently to drugs, which complicates the asthma treatment protocols. Therefore, development of a broadly effective asthma therapeutic measure is needed.

Allergen-specific immunotherapy (ASIT) is the only clinical practice, nowadays, that can lead to the long-term resolution or cure of allergic diseases (9). Successful ASIT leads to several changes in the treated allergic subjects including deviation of the exaggerated type 2 immune response towards the nonpathogenic type 1 response (restoring Th1/Th2 homeostasis); induction of blocking antibodies; decreased pro-inflammatory cytokines release from mast cells, eosinophils, and T cells; decreased recruitment of mast cells, basophils, and eosinophils to the bronchial mucosa after allergen re-exposure; decreased release of mediators from mast cells and basophils; and, most of all, generation of regulatory immune responses. ASIT has shown therapeutic effectiveness for many kinds of allergy, including Hymenoptera stings and allergic rhinitis caused by pollen, house dust mites (HDMs), and pets (9).

Chronically infecting parasites have the capacity to evade/downregulate the host immune responses directed against themselves for their long-term parasitism. One strategy used commonly by many parasites, including *Brugia malayi*—the causative agent of lymphatic filariasis, is the production of immunomodulatory products that induce the generation of regulatory immune cells, especially regulatory T cells (Tregs), regulatory B cells (Bregs), alternatively activated macrophages, and tolerogenic dendritic cells (DCs), to suppress the host effector activities directed against the parasites (10). The host immune suppression or immune unresponsiveness mediated by chronic parasitic infections has a spillover effect that benefits the host by reducing the prevalence of inflammatory disorders including autoimmunity and allergic diseases (10–14). *B. malayi* produces transforming growth factor-β homologue (TGH), which downregulates the host immune response by causing increment of several types of Tregs in the infected hosts, including IL-10-producing Tr1, TGF-β-producing Th3, and FoxP3-expressing peripheral and thymus-derived Tregs (14, 15). In this study, we hypothesized that a measure that could cause an increment of the Treg number and enhancement of their activities in the affected respiratory tissue would be a feasible and broadly effective strategy for the treatment of respiratory inflammation caused by allergens. Thus, the *B. malayi* immunosuppressive protein, TGH, was added to a component-resolved allergen vaccine for treatment of the allergy caused by the American CR (*Periplaneta americana*). Recombinant *B. malayi* TGH was mixed with recombinant Per a 9 which is the *P. americana* major allergen (16), and the vaccine was formulated by entrapping the mixture in cationic liposome. Therapeutic efficacy and molecular mechanisms of the admixed vaccine (liposome-entrapped TGH + Per a 9) in causing mitigation of the allergic manifestations in the mouse model of CR allergy, was investigated in comparison with the liposome-entrapped-Per a 9 alone, liposome-entrapped-TGH alone, and placebo (liposome-entrapped-buffer).

## Materials and Methods

### Chemicals

Hematoxylin & eosin, periodic acid–Schiff (PAS) and Masson’s trichrome (TRI) dyes were from Merck Millipore, USA. Alum adjuvant (Pierce), RevertAid H Minus Reverse Transcriptase, and Rapid DNA Ligation Kit were from Thermo Fisher Scientific, USA. *Limulus* amoebocyte lysate assay kit was from Biolasco, Taiwan. Adenosine assay kit was from Cell Biolabs, USA. Tetanus toxoid (TT) was from Biofarma, Indonesia. Anti-CD16/32 (FcX), anti-CD3-FITC, anti-CD4-PerCP and anti-FoxP3-Alexa Flour^®^647 were from BioLegend, USA. Anti-CD25-BV421 and anti-CD45RA-PE were from BD Biosciences, USA. IDO1 ELISA kit was from LifeSpan Biosciences, USA. Phosphatidylcholine was from Lipoid-AG, Switzerland. Cholesterol was from Sigma-Aldrich, Germany. Dimethyldioctadecyl-ammoniumbromide (DDAB) was from Honeywell Research Chemicals, USA.

### Ethics Statement

All experiments involving animals were conducted according to the ethical policies and guideline of the National Research council of Thailand (NRCT). Animal manipulation was performed by scientist and veterinarian holding certificates for use of experimental animals certified by the NRCT. Mouse experiments were approved by the Care and Use Committee, Faculty of Medicine Siriraj Hospital, Mahidol University, Bangkok (approval no. SI-ACUC 003/2561). Gerbil experiments for adult *B. malayi* preparation was approved by Faculty of Medicine, Chiang Mai University, Thailand (approval no. 05/2558).

### Preparation of Recombinant Per a 9

Per a 9 is a major allergen of *P. americana* that sensitized 100% of the cockroach allergic Thai patients (16); therefore, the allergen was chosen as an allergen vaccine component for CR allergy based on the principle of component-resolved immunotherapy (CRIT) (17). Gene coding for full length Per a 9 was PCR amplified using cDNA synthesized from CRE-total RNA as template and primers designed from GenBank database (AY563004.1): forward-primer: 5’-GGGATCCGATGGTGGACGCCGCA-3’, and reverse-primer: 5’-GCAAGCTTGAGCGAGCTCTCCAG-3’. The PCR reaction mixture was cDNA (1 μl), 10 μM each primer, 25 mM MgCl_2_ (2.5 μl), 2.5 mM dNTPs (2 μl), 5 units/μl DNA polymerase (0.2 μl), and UDW (40.3 μl). The thermal cycles were: denaturation at 94°C for 5 min; 30 cycles of 94°C for 30 s; 50°C for 30 s; and 72°C for 40 s; and a final extension at 72°C for 7 min. The verified PCR product was inserted into the pET23b^+^ vector and the pET23b^+^-*Per a 9* plasmid was transformed into BL21 (DE3) *E*. *coli*. The transformed BL21 (DE3) *E*. *coli* were grown under 0.4 mM isopropyl β- d-1-thiogalactopyranoside (IPTG) induction for 6 h; the bacterial cells were collected after centrifugation (12,000×*g*, 4°C, 30 min). Each 2 g of the *E. coli* wet pellet were lysed with 10 ml of BugBuster™ protein extraction reagent (Novagen, Schwalbach, Germany) containing 20 μl of Lysonase™ bioprocessing reagent (Novagen) and the preparation was centrifuged at 8,000×*g* at 4°C for 30 min. Inclusion body (IB) in the pellet was washed twice with Wash-100 reagent and once with Wash-114 reagent with vigorous shaking and centrifuged. The IB was then washed with Wash-Solvent reagent and Milli Q water on ice also with vigorous shaking and then centrifuged. Five milliliters of buffer [50 mM CAPS, pH 11.0; 0.3% (w/v) *N*-Lauryl sarcosine; and 1 mM DTT] were added to reconstitute 5 mg of purified IB and kept at 4°C for 16 h. The dissolved protein was loaded into the Slide-A-Lyzer^®^ 2K Dialysis Cassettes G2 (Thermo Fisher Scientific), and the cassette was subjected to dialysis against 750 ml of 20 mM imidazole, pH 8.5, supplemented with 0.1 mM DTT (refolding buffer) at 4°C with slow stirring for 16 h. The refolded protein was subsequently dialyzed further against a dialysis buffer (20 mM imidazole without DTT) with slow stirring at 4°C for 6 h. The preparation was filtered through a 0.2-μm low protein binding Acrodisc^®^ Syringe Filter (Pall, NY, USA) and kept in 30°C water-bath for additional 2 h before adding with 60 mM trehalose. Protein and endotoxin contents were determined by Bradford assay and *Limulus* amebocyte lysate assay (Biolasco, Taiwan), respectively.

### Generation of Mouse Model of Cockroach Allergy and Mouse Sample Collection

Mouse model of *P. americana* allergy was prepared as described previously (18). The allergic mice were used subsequently for evaluation of therapeutic efficacies of the allergen vaccines. Male BALB/c mice (6–8 weeks old) were injected intraperitoneally (i.p) with 3 doses of 150 μg of *P. americana* crude extract (CRE) (containing 0.225 μg of Per a 9) mixed 2:1 (v/v) with alum (Thermo Scientific) in phosphate buffered saline solution, pH 7.4 (PBS) on days 0, 7, and 14. On day 21, the mice were challenged intranasally (i.n.) with 100 μg of CRE in 20 μl PBS (10 μl per nostril). On days 23, 25, and 27, the mice were nebulized with aerosolized CRE (10 mg in 10 ml of PBS). All mice were bled on day 28. Specific serum IgE, IgG1 and IgG2 to the CRE and recombinant Per a 9 were determined by ELISA (18, 19). Sham mice were treated similarly as for the CRE-sensitized mice, but with PBS instead of the CRE. Representative mice of both groups, and normal mice (six mice each) were sacrificed and the mouse lungs were used for histopathological and cytokine studies (19). The experimental timeline for mouse sensitization, allergen provocation (nebulization) and allergy model analysis is shown in **Supplementary Figure 1**.

### Assays for Measuring IgE, IgG1 and IgG2a to CRE, Per a 9 and TGH in Mouse Serum Samples

Serum specific IgE to CRE, Per a 9 and TGH were measured by IgE capture assay. Wells of microplates were coated with 1 μg rat anti-mouse IgE (SouthernBiotech, USA) in 100 μl carbonate-bicarbonate buffer, pH 9.6 (coating buffer) at 4°C overnight. All wells were then washed with PBS containing 0.05% Tween-20 (PBST), blocked with 200 μl of 1% bovine serum albumin (BSA) in PBS, and added with individual mouse serum samples (diluted 1:4 in 100 μl PBS). The plates were maintained in a moist chamber at 37°C for 2 h, and washed. For measuring CRE-specific IgE, 1 μg CRE, rabbit anti-CRE (1:1,000), and goat anti-rabbit immunoglobulin-HRP (Southern Biotech, USA; 1:3,000) were added sequentially with incubation and washing with PBST between the steps. For IgE to Per a 9 and TGH, 1 μg of the respective recombinant proteins were added, incubated, washed and added with 1:3,000 HRP conjugated-anti-6× His Tag antibody (BioLegend, USA). ABTS substrate (KPL by SeraCare Life Sciences, Inc., USA) were used for color development. The enzymatic reaction was stopped by adding 100 μl of 1% SDS in distilled water to all wells. OD405nm of each well was determined against blank (PBS instead of the mouse sample) by an ELISA reader.

IgG1 and IgG2a to CRE, Per a 9 and TGH in the mouse serum samples were measured by indirect ELISA. ELISA wells coated individually with 1 μg of the respective antigens in 100 μl of coating buffer were washed with PBST, blocked with BSA, added with individual mouse serum samples (diluted 1:1,000) and kept in a moist chamber at 37°C for 1 h. After washing, goat anti-mouse IgG1-biotin and rat anti-mouse IgG2a-biotin (both from Southern Biotech; 1:5,000) were added to detect IgG1 and IgG2, respectively. Streptavidin–horseradish peroxidase (HRP) conjugate (SouthernBiotech; 1:3,000) and ABTS substrate (KPL) were used for color development with washing between the two steps. The enzymatic reaction was stopped and the OD405nm of each well was determined as for the IgE measurement.

### Analysis of Lung Histopathological Features

Five micrometer-microtome sections (10–25 sections/mouse group) of mouse lung diaphragmatic lobes were fixed and stained with: hematoxylin and eosin (H&E) for microscopic morphology of the bronchiolar epithelium and infiltrated inflammatory cells; periodic acid–Schiff (PAS) for mucus-producing goblet cell enumeration; Masson’s trichrome (TRI) for investigating collagen deposition and lung fibrosis in the subepithelial layer. Histopathologic appearances of the stained lung sections were rated arbitrarily into grades 0–3 as detailed in **Supplementary Table 1**.

### Determination of Cytokine Gene Expressions in Mouse Lungs

Quantification of *IL-4*, *IL-5*, *IL-6*, *IL-13*, *IL-12a* (*p35*), *IL-12b* (*p40*), *IL-17a*, *TNF-α*, *IFN-γ*, *IL-10*, *TGF-β*, and *IL-35* mRNAs in the mouse lungs was performed by quantitative RT-PCR as described previously (19) using PCR primers as shown in **Supplementary Table 2** and house-keeping-β-actin gene mRNA for RNA normalization.

### Preparation of Recombinant *B. malayi*-TGH

Adult *B. malayi* were prepared as described previously (20). Total RNA was extracted from the worms and cDNA was synthesized. The primers for *TGH* amplification designed from GenBank database (AF104016.1) were forward-primer: 5ˊ**-**CGGGATCCGACCACCGCTTGGCACC-3ˊ, and reverse-primer: 5ˊ-AGTGCGGCCGCAGCACAGGCACACCGC-3ˊ. PCR reaction mixture was cDNA (1 μl), 10 μM each primer, 25 mM MgCl_2_ (1.5 μl), 10 mM dNTPs (0.5 μl), 10× *Taq* buffer with KCl (2.5 μl), 5 units/μl DNA polymerase (0.2 μl), and ultrapure-distilled water (UDW) (18.3 μl). Thermal cycles were: denaturation at 95°C, 5 min; 35 cycles of 95°C, 30 s; 52°C,30 s; and 72°C, 1 min; and extension at 72°C, 30 s. The PCR product was run in 1% agarose gel electrophoresis; the DNA amplicon was gel purified and verified by sequencing. The *TGH-*DNA and the pET23b^+^-vector were cut similarly with *Bam*HI and *Not*I endonucleases. The *TGH* fragments were inserted into the cut vector using the Rapid DNA Ligation Kit (Thermo Fisher Scientific). The pET23b^+^*-TGH* plasmid was transformed into JM109 *E*. *coli*, and colonies were screened by PCR using T7 promoter and terminator primers. The verified recombinant plasmids were isolated from the JM109 *E*. *coli* clones and introduced into NiCo21 (DE3) *E*. *coli*. The transformed NiCo21 (DE3) *E*. *coli* were grown under 0.4 mM isopropyl β- d-1-thiogalactopyranoside (IPTG) induction for 6 h; the bacterial cells were collected after centrifugation (12,000×*g*, 4°C, 30 min). Purified recombinant TGH was prepared from the bacterial inclusion body as for the recombinant Per a 9.

### Ability of the Recombinant TGH in Induction of the Regulatory T Cell Generation

The recombinant TGH was tested for ability to induce generation of Tregs before use as a component in an allergen vaccine for treatment of CR allergy. For this experiment, peripheral blood mononuclear cells (PBMCs) of normal male BALB/c mice (6–8 weeks old) were cultured in 96-wells-plates (10^5^ cells/well) in RPMI-1640 medium containing 10% fetal bovine serum, 100 units/ml penicillin, and 100 μg/ml streptomycin. Cells in duplicate wells were added, each with 1 µg recombinant TGH. Positive and negative controls were tetanus toxoid (TT) and the medium alone, respectively. After 24-h incubation, the surface Fc receptors of the cells were blocked by incubating with anti-CD16/32 for 15 min; the cells were washed and stained by anti-CD3-FITC, anti-CD4-PerCP, anti-CD25-BV421, and anti-CD45RA-PE. Intracellular FoxP3 was stained by anti-FoxP3-AlexaFlour^®^647. Each preparation was analyzed for percent CD3^+^CD4^+^CD25^hi^FoxP3^+^CD45RA^–^Tregs by flow cytometry (BD LSRII; BD Biosciences).

### Formulation of Therapeutic Vaccines for Cockroach Allergy Treatment and Placebo

Multilamellar liposome (L) was prepared (11) from phosphatidylcholine (PC) and cholesterol (C) using dimethyldioctadecyl-ammoniumbromide (DDAB) as a cationic surfactant. Briefly, DDAB, PC, and C were mixed (2:1:1) using dichloromethane as solvent. The lipid solution (1 ml) was rotated in a round-bottom flask to obtain a thin film and added to mix with 500 μl PBS containing vaccine components: TGH (1.25 mg, endotoxin content 0.25 EU/μg), Per a 9 (1.25 mg, endotoxin content 0.25 EU/μg), or mixture of TGH (0.625 mg) and Per a 9 (0.625 mg). The formulated liposome-entrapped vaccines were designated L-TGH, L-Per a 9 and L-(TGH + Per a 9), respectively. Placebo was liposome-entrapped PBS (L-P). All the preparations were characterized for zeta potentials, sizes, and polydispersity indices. Percent protein entrapment of each liposomal vaccine was determined (19).

### Immunotherapy of Cockroach Allergic Mice and Vaccine Efficacy Evaluation

Mice allergic to the *P. americana* crude extract (CRE) were divided into four groups (six mice/group). Groups 1–3 were vaccinated i.n. with 20 μl L-TGH, L-Per a 9, and L-(TGH + Per a 9), respectively; group 4 received L-P. Seven booster doses were given to the mice on alternate days (same dose and route as for the priming). Seven days post-last vaccine/placebo booster, the mice of all groups were provoked with aerosolized CRE (10 mg in 10 ml PBS). On the next day, they were bled for measurements of serum specific IgE, IgG1 and IgG2a to CRE, Per a 9 and TGH antibodies and adenosine (one of the immunosuppressive products of Tregs). Thereafter, the mice were sacrificed, and their right lungs were processed for histopathological and cytokine studies. The left lung was used for measuring the level of indoleamine 2,3 dioxygenase-1 (IDO1) which is the signature of tolerogenic dendritic cells (DCs) (19). The experimental timeline of vaccination (immunotherapy) of the CRE-allergic mice, allergen provocation (aerosolized challenge with CRE) and vaccine efficacy analysis (determination of the therapeutic efficacies of vaccines after vaccination and CRE-provocation) is shown in **Supplementary Figure 2**.

### Measurement of Mouse Serum Adenosine and Lung IDO1

Levels of adenosine in the mouse sera were measured using an Adenosine Assay Kit (Cell Biolabs, USA). A reaction mixture was prepared by mixing fluorometric probe (1:100), avidin-horseradish peroxidase (1:500), adenosine deaminase (1:500), purine nucleoside phosphorylase (1:10), and xanthine oxidase 1:50 in assay buffer. A control mixture containing the same contents as for the reaction mixture except for adenosine deaminase was prepared also. Adenosine standard solutions and mouse samples (50 μl) were added individually into the wells of a black microtiter plate; then either 50 μl of the reaction or a control mixture was added in duplicate. The plate was incubated (37°C, 15 min) in darkness. The excitation (530–570 nm) and emission (590–600 nm) of each well’s content was determined using a fluorescence microplate reader (BioTekTM SynergyTM H1 Hybrid Multi-Mode Monochromater Fluorescence Microplate Reader). The net relative fluorescence unit (RFU) of each sample (difference between the RFU of the wells added with reaction-mixture and the well added with control mixture of the same sample) was used to extrapolate the adenosine level in the mouse serum from the standard adenosine RFU curve.

The levels of mouse lung IDO1 were measured as described previously (19) using IDO1-ELISA kit (LifeSpan Biosciences, USA).

### Data Analysis

SPSS statistics version 17.0 (SPSS, USA) was used. The mean ± SD of the antibody levels, tissue histopathology, cytokine expression data, and levels of serum adenosine and lung IDO1 were analyzed using ANOVA followed by post-hoc comparison using the least significant difference (LSD) and independent *t*-test. *P <*0.05 was statistically significant.

## Results

### Recombinant Per a 9

The 6**×**-His tagged-Per a 9 (38–40 kDa) were purified from the lysate of pET23b^+^-*Per a 9* plasmid transformed-BL21 (DE3) *E*. *coli* using Ni-NTA affinity resin. The recombinant protein after SDS-PAGE and CBB dye staining is shown in **Supplementary Figure 3A**; the respective Western blot pattern (probed with anti 6**×**-His antibody) is shown in **Supplementary Figure 3B**. The protein was also verified by LC-MS/MS as *P. americana* Per a 9 (data not shown). Endotoxin contents in the recombinant Per a 9 was 0.25 EU/μg.

### Mouse Model of Cockroach Allergy

**Figure 1:**
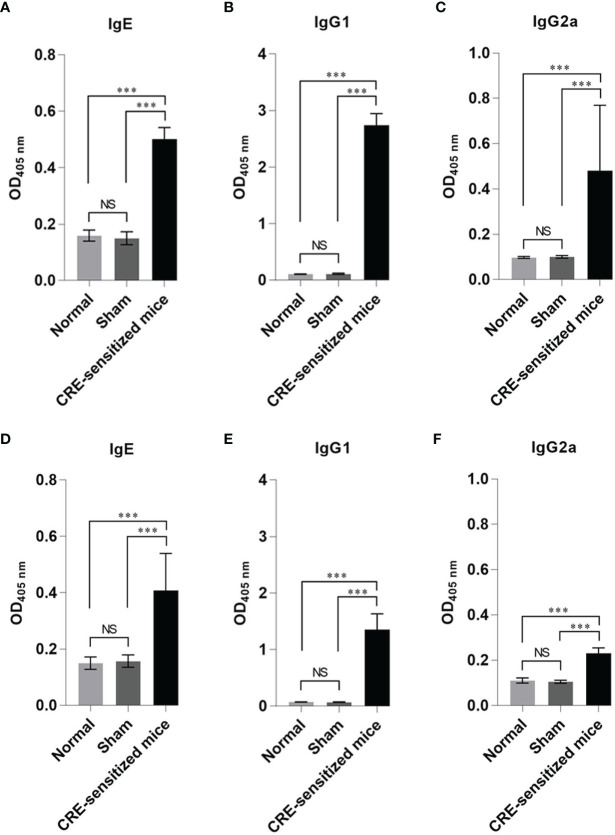
Optical density (OD) 450 nm (mean ± SD) of specific antibodies in the serum samples of the CRE-sensitized, sham, and normal mice. **(A–C)** are IgE, IgG1 and IgG2 to CRE, respectively. **(D–F)** are IgE, IgG1 and IgG2a to recombinant Per a 9, respectively. The CRE-sensitized mice had significantly higher serum specific antibodies to CRE and recombinant Per a 9 than the sham and normal mice. The antibody levels of sham and normal mice were not different statistically. NS, not significantly different; ***, *p* < 0.001.

The mean ± SD of specific IgE, IgG1, and IgG2a to CRE and recombinant Per a 9 in sera of the CRE-sensitized, sham, and normal mice are shown in **Figure 1**. The mean ± SD of CRE-specific IgE, IgG1, and IgG2a of the CRE-sensitized mice were significantly higher than those of the sham and normal mice, whereas the CRE-specific IgE, IgG1, and IgG2a of the normal and sham mice were not statistically different (**Figures 1A–C**). The mean ± SD of Per a 9-specific IgE, IgG1, and IgG2a of the CRE-sensitized mice are shown in **Figures 1D–F**, respectively. The CRE-sensitized mice showed similar trends of serum antibody levels to the Per a 9 as for those to the CRE, albeit lower levels.

**Figure 2:**
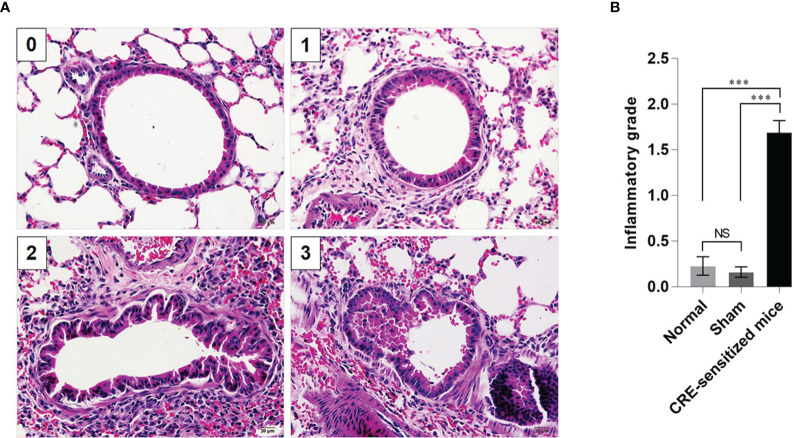
Histopathological appearance of mouse lung sections stained with hematoxylin and eosin (H&E) dyes. **(A)** Inflammatory grades 0–3 (see **Supplementary Table 1**). **(B)** Average inflammatory grades of the lungs of the CRE-sensitized, sham, and normal mice. NS, not significantly different; ***, *p* < 0.001.

**Figure 3:**
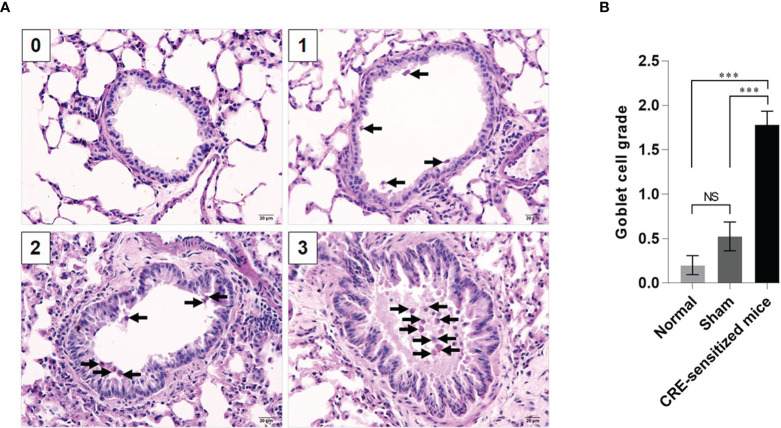
Histopathological appearance of mouse lung sections stained by periodic acid–Schiff (PAS) dye to reveal goblet cells (black arrows). **(A)** Goblet cell grades 0–3. **(B)** Average goblet cell grades of the normal, sham and CRE-sensitized mice. NS, not significantly different; ***, *p* < 0.001.

**Figure 4:**
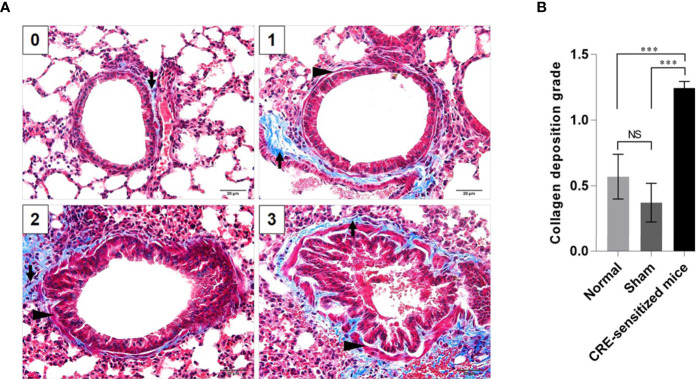
Histopathological appearance of mouse lung sections revealed by Masson’s trichrome (TRI) staining. **(A)** Grades 0–3 of collagen deposition and fibrotic change in submucosa. The collagen is stained blue (black arrows) and smooth muscle is stained red (black arrowheads) by the TRI. **(B)** Average grades of the collagen deposition and fibrotic change in subepithelial layer of the small brionchioles of the CRE-sensitized, sham, and normal mice. NS, not significantly different; ***, *p* < 0.001.

Grades 0–3 of mouse lung inflammatory features stained by H & E, PAS and TRI are shown in **Figures 2A**, **3A** and **4A**, respectively. The average grade of lung inflammation (infiltrated inflammatory cells and histopathological features) of the CRE-sensitized mice was significantly higher than the inflammatory grades of the sham and normal mice (**Figure 2B**). The inflammatory cells around the bronchioles were neutrophils (predominant), lymphocytes, eosinophils and mast cells. The mean goblet cell grade of the CRE-sensitized mice was significantly higher than in the sham and normal mice (**Figure 3B**). Likewise, the average grade of collagen deposition and fibrotic change in the CRE-sensitized mice lungs was significantly higher than in the sham and normal mice (**Figure 4B**). The grades of lung inflammation, goblet cell numbers and submucosal features of sham and normal mice were not significantly different.

**Figure 5:**
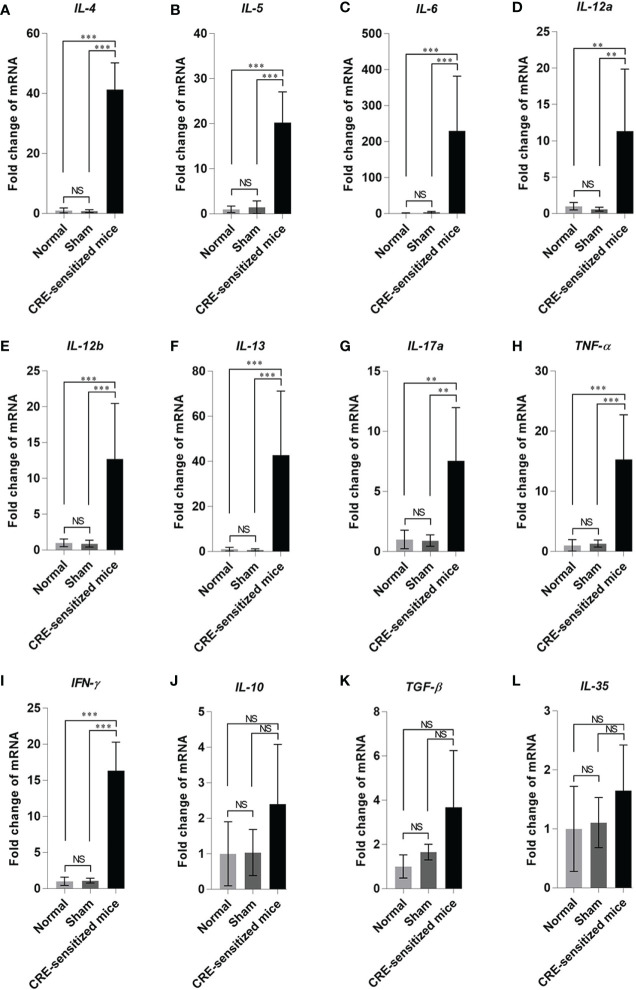
Fold changes of cytokine gene expressions: **(A)** *IL-4*, **(B)** *IL-5*, **(C)** *IL-6*, **(D)** *IL-12a*, **(E)** *IL-12b*, **(F)** *IL-13*, **(G)** *IL-17a*, **(H)** *TNF-α*, **(I)** *IFN-γ*, **(J)** *IL-10*, **(K)** *TGF-β*, and **(L)** *IL-35* in the lung tissues of the CRE-sensitized, sham, and normal mice. NS, not significantly different; **, *p* < 0.01; ***, *p* < 0.001.

The fold increase in lung cytokine gene mRNAs of the CRE-sensitized, sham, and normal mice, compared to the housekeeping β-actin mRNA are shown in **Figure 5**. The CRE-sensitized mice had an upregulation of *IL-4*, *IL-5*, *IL-6*, *IL-12a*, *IL-12b*, *IL-13*, *IL-17a*, *TNF-α*, *IFN-γ*, *IL-10*, and *TGF-β* compared to the sham and normal mice.

It can be concluded from the data of serum-specific IgE, lung histopathological grades, and lung cytokine expression profiles that the CRE-sensitized mice were allergic to the *P. americana* crude extract.

### Recombinant *B. malayi* TGH and Its Ability to Induce Treg Generation *In Vitro*

The recombinant 6× His tagged-TGH of *B. malayi* were purified from the lysate of pET23b^+^*-TGH* plasmid-transformed NiCo21 (DE3) *E*. *coli* by using Ni-NTA column. **Supplementary Figure 4** shows SDS-PAGE and Western blot patterns of recombinant TGH (~38 kDa) after staining with CBB and probing with anti-6× His antibody, respectively. The protein was also verified by LC-MS/MS as *B. malayi* TGH (data not shown). Endotoxin content of the recombinant TGH was 0.25 EU/µg.

**Figure 6:**
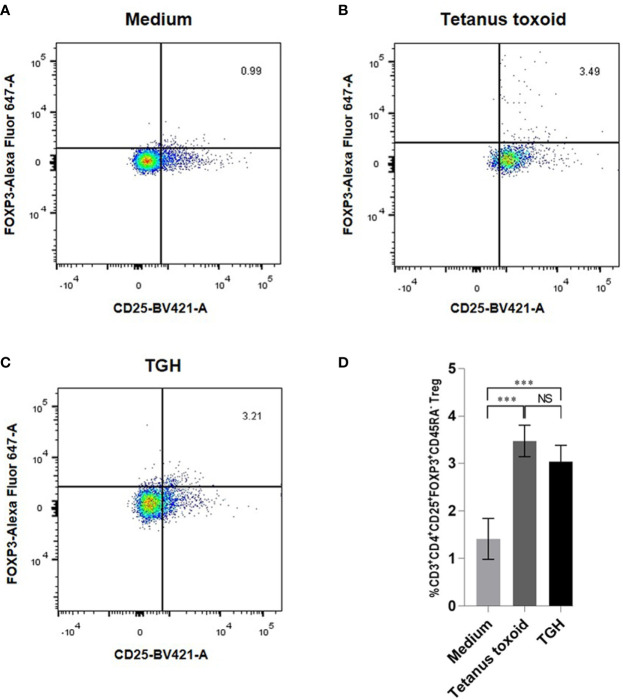
Percentages of CD3^+^CD4^+^CD25^hi^FoxP3^+^CD45RA^–^ Tregs of the mouse PBMCs cultured for 24 h with: **(A)** medium alone, **(B)** tetanus toxoid, and **(C)** TGH. **(D)** Comparison of the percentages of Tregs among the treatment groups. NS, not significantly different; ***, *p* < 0.001.

The percentages of CD3^+^CD4^+^CD25^hi^FoxP3^+^CD45RA^–^ Tregs in PBMCs cultured in the medium with recombinant TGH, medium alone (negative control), medium with tetanus toxoid (positive control) were 3.07, 1.45, and 3.4%, respectively (**Figure 6**). The percent Tregs in the PBMC co-cultured with tetanus toxoid and recombinant TGH were not different statistically, indicating that the recombinant *B. malayi* TGH has inherent property of the native counterpart in inducing Treg generation. Therefore, the recombinant TGH was used further as a component in vaccines for treatment of the CRE-allergic mice.

### Characteristics of the Therapeutic Vaccines and Placebo

The characteristics of the liposome micelles of the three vaccines, i.e., liposome-entrapped TGH and Per a 9 mixture (L-TGH-Per a 9), L-TGH alone, L-Per a 9 alone, and placebo are shown in **Table 1**. All micelles were larger than 3,000 nm, carried positive charge, and were relatively homogeneous. The vaccines contained more than 85% of entrapped-antigens.

**Table 1:** Characteristics of the liposome-entrapped vaccines and placebo.

Parameter	Vaccine			Placebo (L-P)
	L-TGH	L-Per a 9	L-TGH + Per a 9	
Size (nm)	3,539.82 ± 189.24	5,744.67 ± 340.65	3,985.00 ± 243.01	4,519.33 ± 191.11
Polydispersity index	0.17 ± 0.08	0.20 ± 0.18	0.42 ± 0.24	0.49 ± 0.01
Zeta potential (mV)	21.38 ± 0.19	26.40 ± 0.36	18.35 ± 0.23	26.73 ± 0.12
Immunogen entrapment (%)	90.75	89.63	87.36	NA

NA, not applicable.

### Efficacy of the Vaccines Against Cockroach Allergy

**Figure 7:**
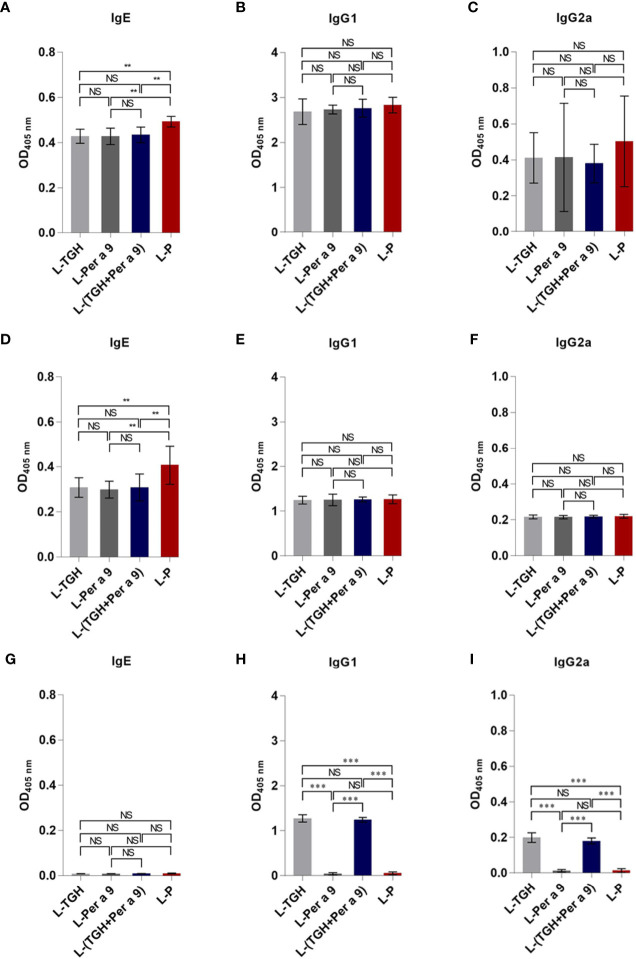
ELISA OD 405 nm (mean ± SD) of specific antibodies in sera of the CRE-allergic mice treated with L-TGH, L-Per a 9, and L-(TGH + Per a 9) vaccines compared to the placebo (L-P). **(A–C)** are IgE, IgG1, IgG2a to CRE; **(D–F)** are IgE, IgG1, IgG2a to recombinant Per a 9; **(G–I)** are IgE, IgG1, IgG2a to recombinant TGH. NS, not significantly different; **, *p* < 0.01; ***, *p* < 0.001.

Levels of specific IgG1, and IgG2 to CRE in the CRE-allergic mice after treatment with the three vaccines and placebo were not different (**Figures 7B, C**). Specific IgE to CRE and recombinant Per a 9 of the allergic mice after treatment with the three vaccines was significantly reduced compared to the mice treated with L-P (**, *p <*0.01) (**Figures 7A, D**). The IgE to recombinant Per a 9 in allergic mice treated with L-TGH + Per a 9 and L-TGH were not different (**Figures 7A, D**). The IgG1 and IgG2 to recombinant Per a 9 in all mouse groups were not different (**Figures 7E, F**, respectively**)**. The IgE antibodies to recombinant TGH in allergic mice treated with all vaccines and placebo were negligible (**Figure 7G**). Allergic mice treated with L-TGH and L-TGH + Per a 9 had significant IgG1 and IgG2 responses to recombinant TGH compared to the L-Per a 9 and L-P groups which the IgG1 and IgG2 levels to the TGH were negligible (**Figures 7H, I**).

**Figure 8:**
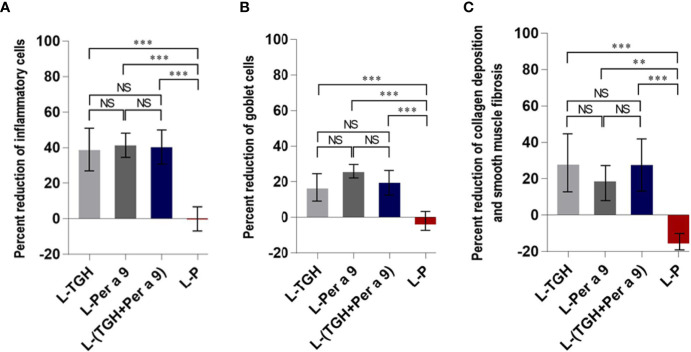
Percent reduction in histopathology grades (observed at 400× original magnification) in the lungs of the CRE-allergic mice after treatment with the vaccines compared to the placebo. **(A)** Inflammatory grades, **(B)** Goblet cell grades, and **(C)** Collagen deposition and smooth muscle fibrosis grades. NS, not significantly different; **, *p* < 0.01; ***, *p* < 0.001.

**Figure 9:**
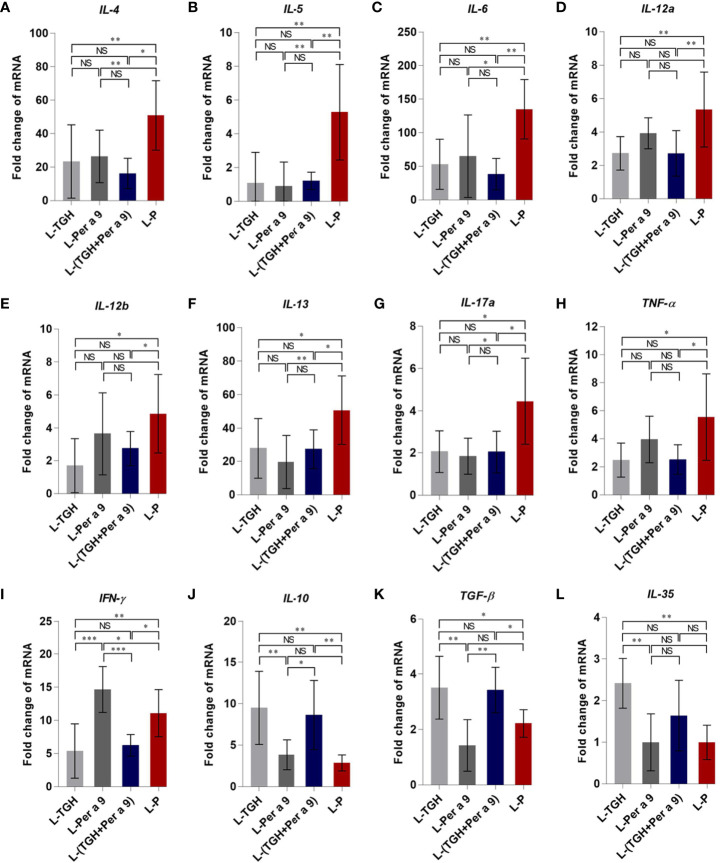
Expression of cytokine genes in the lungs of the CRE-allergic mice after treatment with the L-TGH, L-Per a 9, and L-(TGH + Per a 9) vaccines and the placebo (L-P). **(A)** *IL-4*, **(B)** *IL-5*, **(C)** *IL-6*, **(D)** *IL-12a*, **(E)** *IL-12b*, **(F)** *IL-13*, **(G)** *IL-17a*, **(H)** *TNF-α*, **(I)** *IFN-γ*, **(J)** *IL-10*, **(K)** *TGF-β*, and **(L)** *IL-35*. NS, not significantly different; *, *p* < 0.05; **, *p* < 0.01; ***, *p* < 0.001.

All of the three vaccines could cause significant reduction of histopathological grades in lungs, *i.e.*, the inflammation, the goblet cell grades, and the collagen deposition and fibrosis of the vaccine-treated allergic mice compared to the placebo mice (**Figure 8**). Allergic mice treated with L-TGH, L-Per a 9, and L-(TGH + Per a 9) had lower expressions of *IL-4*, *IL-5*, *IL-6*, *IL-13*, and *IL-17a* than placebo mice (**Figure 9**). The L-TGH and L-(TGH + Per a 9) also caused lower expressions of *IL-12a*, *IL-12b*, *TNF-α*, and *IFN-γ*, but an upregulation of *TGF-β* and *IL-10* of the vaccinated mice compared to placebo mice. Expression of IL-35 of the L-TGH treated-allergic mice was higher than in the L-Per a 9 and L-P mouse groups but not different from L-(TGH + Per a 9) treated mice. Unlike the L-TGH, L-(TGH + Per a 9) and placebo-treated mice, the allergic mice treated with L-Per a 9 had an upregulation of *IL-12a*, *IL-12b*, and *IFN-γ*, but did not have upregulation of immunosuppressive cytokine genes.

**Figure 10:**
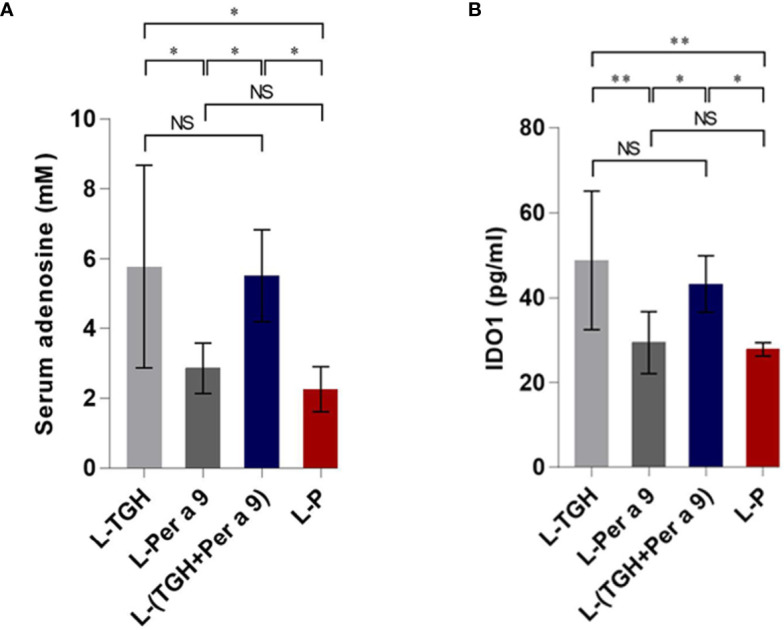
Levels of serum adenosine and lung IDO1 of the vaccinated allergic and placebo mice. **(A)** Serum adenosine. **(B)** Lung IDO1. NS, not significantly different; *, *p* < 0.05; **, *p* < 0.01.

The levels of serum adenosine and lung IDO1 of the vaccine-treated allergic mice compared to the placebo mice are shown in **Figure 10**. Only the L-TGH and L-(TGH + Per a 9) vaccines caused a significant increase in serum adenosine and lung IDO1 in the treated mice.

## Discussion

For testing therapeutic efficacies and molecular mechanisms of the allergen vaccines against cockroach allergy that were formulated in this study, a mouse model of American cockroach allergy was generated. BALB/c mice were used for the model because this mouse strain has inherent robust IL-4 production, *i.e.*, they have a propensity to develop a vigorous type 2 response that resembles many features, although not all, of human asthma (21). By using the same protocol as reported previously (18), the mouse model of American cockroach allergy was generated, indicating reproducibility of the protocol. We chose to prime naïve mice by injecting an alum–CRE mixture intraperitoneally; as alum is an adjuvant of the type 2 response, which does not typically enhance CD8 T-cell responses (22). APCs in the mouse peritoneal cavity constitutively expressed co-stimulatory molecules, CD40 and B7, and MHC class-II molecules, indicating that the cells were more mature and ready to present antigen to T cells than their respiratory tract counterparts (23). The allergen boosters were given intranasally, so cells at the upper respiratory lymphoid tissue (immune inductive site) of the primed mice could be stimulated; then the activated lymphocytes would leave the inductive site, circulate, and home in the lower respiratory lymphoid tissues (immune effector site), based on the mucosal immune system common traffic (24).

The CRE-sensitized mice showed a significant rise in serum-specific IgE to CRE and Per a 9 contained in the CRE; upregulation of type 2 cytokine genes, e.g., *IL-4*, *IL-5*, *IL-10*, and *IL-13*; airway inflammation; and tissue remodeling. They also showed an upregulation of *IL-6*, *IL-12a (p35)*, *IL-12b (p40)*, *IL-17A*, *TNF-α*, and *TGF-β*. In mice, *IL-6*, *TGF-β*, and IL-23 cytokines (and RORγt transcription factor) are critical factors for Th17 development (25–27). Although the expression of p19 gene of IL-23 was not determined in this study, the increased mRNAs of *IL-12a* (encodes p35 shared by IL-12 and IL-23), *IL-6*, *TGF-β*, and *IL-17a* indicated that besides Th2 effector cells, the Th17 cells were generated also in the CRE-sensitized mice. Two functionally different Th17 populations can be generated in the mice. IL-6 and TGF-β could induce the generation of regulatory Th17 cells that play a protective role for lung inflammation, while Th17 cells generated under the influence of IL-6 and IL-23 are pathogenic effector Th17 cells (28). The pathogenic Th17 cells differentiated in chronically inflamed allergic airway inflammation could abrogate regulatory T-cell-mediated tolerance and contribute to airway remodeling (29). The elevation of IL-17A (the principal pro-inflammatory cytokine of pathogenic Th17 cells) has been found in severe airway inflammation and asthma. IL-17A acts *via* p38 MAPK to increase the stability of TNF-α; then the TNF-α induces the expression of a CXC-chemokine, IL-8 (30). IL-8 is a potent neutrophil chemoattractant that causes neutrophil influx to the inflamed lung (31), which concords with our finding that the inflammatory cells in the CRE-sensitized mouse lungs were predominantly neutrophils; the other infiltrated cell types were lymphocytes, eosinophils and mast cells. Increased expressions of *IL-12a*, *IL-12b*, and *IFN-γ* in the CRE-sensitize mice indicated that the mice had also the type 1 immune response, which should indicate an attempt of the immune system to maintain Th1/Th2 homeostasis (striking a balance of types 1 and 2 responses). The CRE-sensitized mice also had high serum-specific IgG1 and IgG2a responses to CRE and the Per a 9. The role of different IgG subtypes (including IgG4 in humans) in allergies is still controversial. The IgG antibody’s roles seem to depend on whether the Fcγ receptors that they fixed on the immune cells are receptors for activation (FcγRI/CD64, FcγRIII/CD16, and FcγRIV/CD16-2) or inhibition (FcγRIIB/CD32B) (32). Thus, increased IgG1 and IgG2a to CRE and Per a 9 in the CRE-sensitized mice may have either an inflammatory or anti-inflammatory role, depending on the nature of the FcγR signaling pathway of the APCs involved. Taken altogether, the CRE-sensitized mice had the immunological signatures of type 2 inflammation, *i.e.*, they were allergic to CRE. Therefore, the remaining CRE-allergic mice were used further in the experiments for evaluation of the therapeutic efficacy of the three vaccines.

Natural regulatory T cells are produced in the thymus during the negative selection of T cells (33). Tregs can also be induced from naive T cells in the periphery, called inducible Tregs (iTregs) (33). Usually, Tregs maintain immunological unresponsiveness/tolerance to self-antigens and suppress excessive immune responses that are hazardous to the host, including allergic inflammation (34). Data in the literature showed that during eosinophilic and neutrophilic inflammations from CR-allergy, 30 and 10%, respectively, of the CD4^+^ T cells that infiltrated into lungs were FoxP3^+^, which is one of the signatures of potent immune suppression (35). Although the infiltrated Tregs play a role in controlling lung inflammation (36), their activity can be compromised during allergic inflammation rendering them inability to cope with the exaggerated type 2 inflammatory response in the affected lungs (35). Thus, a measure that could cause Treg number increment and enhancement of their activities in the affected respiratory tissue should bring about mitigation, if not abrogation of the respiratory inflammation.

Previous data demonstrated that co-incubation of human Treg-specific epitopic peptide, called “Tregitope” derived from immunoglobulin G, and HDM lysate could suppress cytokine secretion in PBMCs from HDM-allergic individuals (37). Because the *B. malayi* parasite produces the immunosuppressive protein, i.e., TGH, which can induce the generation of many kinds of iTregs (14, 15), we envisaged that a vaccine made of CR-allergen mixed with *B. malayi* TGH and administered intranasally to CR-allergic hosts should induce the generation of Tregs that home in the lower respiratory tract, where they can exert broadly effective immunosuppressive/anti-inflammatory activities. Consequently, we first tested whether the recombinant *B. malayi* TGH protein would induce Treg generation like its native counterpart or not. Reproducible experiments demonstrated that the recombinant *B. malayi* TGH could induce the generation of CD3^+^CD4^+^CD25^hi^FoxP3^+^CD45RA^−^ Tregs at a similar magnitude as the positive control (tetanus toxoid), indicating that the recombinant TGH has the inherent immunosuppressive property of its native counterpart. Therefore, the active recombinant TGH was used instead of the native TGH in formulating the therapeutic vaccines for CR-allergy, as the former would be more easily, readily and adequately available in the future. Based on the principle of a component-resolved immunotherapy (CRIT) system (17, 38), Per a 9 was chosen as the vaccine component for treatment of the CR-allergy because this protein is the major *P. americana*-allergen that reacted with the serum samples of all the tested CR-allergic Thai patients (16). The liposome that we used served not only as the vaccine delivery vehicle, but also as an adjuvant of the cell-mediated immune response (39). The micelles of all the vaccine and placebo preparations carried positive charge. They were relatively homogeneous as indicated by their low polydispersity indices. They were all larger than 3,000 nm. By carrying a positive surface charge, the micelles can coalesce well with the negatively-charged plasma membrane of the encountering APCs, such that the liposome-entrapped antigen(s) could be delivered directly into the cytoplasm, where they would be processed by the cytosolic pathway and presented to the cognate T cells *via* MHC class 1 molecules. Even though some of the micelles might be endocytosed, they should be retained in the early endosome owing to their large size (larger than 200 nm), where the antigen would be processed and presented as a type 1 response rather than type 2 (40). The liposome used in this study was made of phosphatidylcholine and cholesterol, as both lipids are normal components of the plasma membrane and so the liposome should be biocompatible to mammalian hosts.

The CRE-allergic mice treated with L-TGH, L-Per a 9, and L-(TGH + Per a 9) did not show a significant difference in serum specific IgG1 and IgG2 antibodies to CRE, Per a 9 and TGH compared to the placebo mice. However, the levels of CRE and Per a 9 specific IgE were reduced in the vaccine-treated mice compared to L-P group. Data on the specific antibodies of the vaccinated allergic mice agreed with previous findings of ASIT for CR and HDM allergies (9, 18, 19). Different outcomes in serum specific-IgE response after ASIT completion have been documented: some studies revealed no change, while others showed an increase or decrease in IgE levels (41, 42). The levels of serum-specific IgE decreased over time in grass pollen ASIT (43, 44). The IgE levels rose shortly after successful immunotherapy, and later reduced, although the reduced levels did not go below pretreatment levels (42). The different outcomes of ASIT on the antibody responses observed in different studies, including this study, may depend on the study duration, amount of vaccine component per dose, number of vaccine doses, immunization schedule, sampling time, and especially on the types of allergies, if not also the host genetics. For our study, significant reduction of IgE to CRE and Per a 9 was observed. The TGH is a nematode antigen and could, in principle, induce an allergic immune response in the animals during the vaccination against the allergen. The finding that the antibody responses in the mice treated with vaccines containing TGH were negligible and not different from the other mouse groups, indicating that the TGH is not an allergen (naturally, it is an immunosuppressive protein).

The CRE-allergic mice treated with the three vaccines in this study had significant reduction of lung inflammatory cells, goblet cells, and submucosal collagen deposition and fibrosis, compared to the placebo mice. The vaccine-treated mice had reduced mRNAs of *IL-4*, *IL-5*, *IL-6*, *IL-13*, and *IL-17a*, indicating vaccines-mediated suppression of type 2 and Th17 responses. The L-Per a 9-vaccinated mice had high expression of *IFN-γ* mRNA compared to the other mouse groups, implying that this therapeutic vaccine could cause a replacement of the pathogenic type 2 by the type 1 response. IFN-γ, the principal type 1 cytokine, could counteract and attenuate the type 2 response of allergic disease (45). The molecular mechanisms of IFN-γ on the resolution/attenuation of allergy-associated immunopathology have been reviewed (45). IFN-γ levels were found to be associated with allergy resolution, while lower IFN-γ production in infants with bronchiolitis was an indicator of lower pulmonary function, which was related to the later development of asthma (patients with severe asthma were found to have reduced IFN-γ production in response to allergens) (46). IFN-γ can reinforce the differentiation of naïve T cells to Th1, but not to the Th2 phenotype; inhibit Th2 cell proliferation, recruitment, and differentiation; inhibit eosinophil recruitment to the inflammation site; downregulate IL-4 and GM-CSF production and allergen presentation to T lymphocytes; block IgE isotype switch in B cells; and induce IDO1 expression and nitric oxide production.

The CRE-allergic mice treated with L-TGH and L-(TGH + Per a 9) had a significant reduction of lung histopathology and reduced expressions of Th1, Th2, and Th17 cytokine genes compared to the placebo mice, indicating that the vaccines effectively suppressed lung inflammation in the vaccinated mice. The vaccinated mice also had high expressions of immunosuppressive cytokines IL-10 and TGF-β (also IL-35 for the L-TGH-treated mice). These findings indicate that the L-TGH and L-(TGH + Per a 9) could induce the generation of Tregs (probably also Bregs), which counteract the effector T cells. The molecular mechanisms of Tregs in the suppression of immune responses have been extensively reviewed (47). Tregs can suppress effector cells by many different ways, both directly and indirectly. The suppressive mechanism used by the Tregs depends on the cell milieu, the type of the Tregs, and the status of the target cells (48). Tregs may cause the apoptosis of targets upon cell-to-cell contact by perforins and granzyme-B. Tregs express abundant IL-2 receptor (CD25), which binds IL-2 with high affinity for their growth, survival, and effector differentiation, leaving the IL-2 deprived-effector T cells to die by neglect (a deletional mechanism of peripheral tolerance). Tregs inhibit IL-2 production by polyclonal T cells. They also use their surface ectonucleases (CD39 and CD73) to produce immunosuppressive adenosine from ATP, or transfer cAMP to effector T cells, and suppress TCR-induced Ca2^+^, NF-AT, and NF-κB signaling. Tregs produce IL-10, TGF-β, and IL-35 for inhibiting the activities of a variety of cell types, including effector CD4^+^ and CD8^+^T cells. Tregs can suppress effector T cells indirectly by downregulating the costimulatory molecules CD80/CD86 and MHC molecules on dendritic cells (DCs) *via* CTLA-4, which weakens the T-cell-stimulating activity of the DCs; the DCs thus become “Tolerogenic DCs”. The generated tolerogenic DCs produce indoleamine 2,3 dioxygenase (IDO), which catabolizes tryptophan *via* the kynurenine pathway, leading to a reduced local tryptophan concentration, and elevation of the immunomodulatory tryptophan metabolites, e.g., kynurenic acid, which contributes to the resolution of inflammation and establishment of an immunosuppressive environment (48). Although we did not determine the numbers of regulatory lymphocytes in the lungs of the experimental mice due to the scarcity of mouse lung cells, the findings that adenosine (effector molecule of the Tregs) and IDO1 (signature of tolerogenic DCs) in the lungs of allergic mice treated with L-TGH and L-(TGH + Per a 9) were significantly higher than those in the L-Per a 9 and placebo groups, indicating that the L-TGH and L-(TGH + Per a 9) could effectively induce the generation of regulatory cells in the vaccinated mice, which created a state of immune tolerance.

The generation of iTreg is Treg epitope (Tregitope)-specific, but the immunosuppressive factors/mechanisms produced/mediated by the so-generated iTregs to suppress effector cells act non-specifically (bystander regulation/suppression) (49). L-TGH and L-(TGH + Per a 9) could cause an immunosuppressive environment in the vaccinated mouse lungs; thus, both vaccines have therapeutic potential for not only CR-allergy, but also for other inhalant allergies. Nevertheless, L-(TGH + Per a 9) should confer a more practicality than L-TGH for further clinical application. The TGH in the admixed vaccine causes the generation of TGH-specific Tregs. These iTregs not only tightly regulate the effector cells, but also their suppressive cytokines (IL-10, TGF-β, and/or IL-35) have the ability to convert Per a 9-specific effector T cells directly or indirectly (via tolerogenic DCs) to Per a 9-specific Tregs, through the phenomenon called “Infectious tolerance” (50). Re-exposure naturally to the CR-allergen would act as a booster for reactivation/recall of the Per a 9-specific memory Tregs to exert immunosuppressive activities in tissues. On the contrary, CR-allergen re-exposure should not be able to boost/recall the TGH-specific memory Tregs, which means that in order to maintain the respiratory tolerance, TGH must be re-administered to the patients at some (unknown) intervals, which would not be practical.

In conclusion, the novel intranasal vaccine containing the mixture of major American cockroach allergen (Per a 9) and *B. malayi* immunosuppressive TGH protein effectively suppressed the immunopathology induced by the cockroach allergens. The vaccine should be tested further towards a clinical use as a broadly effective biologic against inhalant allergies.

## Data Availability Statement

The original contributions presented in the study are included in the article/**Supplementary Material**. Further inquiries can be directed to the corresponding author.

## Ethics Statement

The animal study was reviewed and approved by the Care and Use Committee, Faculty of Medicine Siriraj Hospital, Mahidol University and Faculty of Medicine, Chiang Mai University.

## Author Contributions

NS, AT, and WC conceived the project and provided resources. PP did most experiments and prepared figures, UC supervised PP on histopathology, WS helped PP on recombinant protein production and purification, AS prepared adult *Brugia malayi*, OR identified proteins by LC-MS/MS, and KM supervised PP on flow cytometric analysis. NS and WC wrote the manuscript. All authors contributed to the article and approved the submitted version.

## Funding

This study was co-funded by the Thailand Research Fund (RSA6280100) to NS and the NSTDA Chair Professor grant (P-1450624) of the National Science and Technology Development Agency (NSTDA) funded by the Crown Property Bureau of Thailand to WC. PP received the Royal Golden Jubilee PhD scholarship from the Thailand Research Fund.

## Conflict of Interest

The authors declare that the research was conducted in the absence of any commercial or financial relationships that could be construed as a potential conflict of interest.
