# Supplementary material for: A Component-Resolved Therapeutic Vaccine for Cockroach Allergy Made of Per a 9 and Transforming Growth Factor-β Homologue, an Immunosuppressive Protein of Brugia malayi
Source: Front Immunol. 2021 May 31;12:676558. doi: 10.3389/fimmu.2021.676558 (PMC8201610; doi:10.3389/fimmu.2021.676558)
Supplement: Supplementary file 1 [file DataSheet_1.pdf]

Supplementary Material

A component-resolved therapeutic vaccine for cockroach allergy made of Per a 9 and transforming growth factor-β homologue, an immunosuppressive protein of *Brugia malayi*

Pannathee Prangtaworn[1,2], Kodchakorn Mahasongkram[2], Atiporn Saeung[3], Urai Chaisri[4], Watee Seesuay[2], Onrapak Reamtong[5], Anchalee Tungtrongchitr[2], Wanpen Chaicumpa[2], Nitat Sookrung[2,6,*]

[1] Graduate Program in Immunology, Department of Immunology, Faculty of Medicine Siriraj Hospital, Mahidol University, Bangkok 10700, Thailand

[2] Center of Research Excellence on Therapeutic Proteins and Antibody Engineering, Department of Parasitology, Faculty of Medicine Siriraj Hospital, Mahidol University, Bangkok 10700, Thailand

[3] Department of Parasitology, Faculty of Medicine, Chiang-Mai University, Chiang-Mai 50200, Thailand

[4] Department of Tropical Pathology, Faculty of Tropical Medicine, Mahidol University, Bangkok 10400, Thailand

[5] Department of Tropical Molecular Biology and Genetics, Faculty of Tropical Medicine, Mahidol University, Bangkok 10400, Thailand

[6] Biomedical Research Incubation Unit, Department of Research, Faculty of Medicine Siriraj Hospital, Mahidol University, Bangkok 10700, Thailand

Supplementary Table 1. Grades and histological features of mouse lung sections stained with hematoxylin and eosin (H&E), periodic acid–Schiff (PAS), and Masson's trichrome (TRI) dyes.

Grade	Histological features revealed by H&E staining
0	Bronchiole with single layer of ciliated epithelial cells, negligible inflammatory cell infiltration into peribronchiolar tissue, submucosa containing normal smooth muscle cells
1	Thickened bronchiole with ciliated pseudo-stratified columnar epithelial cells, few inflammatory cells in the interstitial area, mild degeneration of smooth muscle cells
2	Bronchiolar epithelial hyperplasia, 2–3 layers of inflammatory cells around the bronchiole, degeneration and detachment of smooth muscle in the submucosal area
3	Epithelial hyperplasia and sloughing, intense inflammatory cell infiltration, necrosis of smooth muscle cells and blockade of the airway
**Grade**	**Histological features revealed by PAS staining**
0	No goblet cells
1	1–5 goblet cells
2	6–20 goblet cells
3	More than 20 goblet cells
**Grade**	**Histological features revealed by TRI staining**
0	Normal collagen content of alveolar septa and thin-walled alveoli
1	Mild collagen deposition and thick fibrosis of the alveolar septa
2	Moderate collagen deposition and continuous fibrosis of the alveolar septa
3	Excessive collagen deposition leading to thickened alveolar septa, compressed alveoli, confluent fibrotic mass, and fibroplasia

Supplementary Table 2. Oligonucleotide primers used in qRT-PCR for monitoring cytokine gene expression.

Gene	Primer sequence	Size of PCR product (bp)
IL-4	F: 5′-TCGGCATTTTGAACGAGGTC-3′	216
	R: 5′-GAAAAGCCCGAAAGAGTCTC-3′	
IL-5	F: 5′-ATGATCGTGCCTCTGTGCCTGGAGC-3′	242
	R: 5′-CTGTTTTTCCTGGAGTAAACTGGGG-3′	
IL-6	F: 5’-TCCAGTTGCCTTCTTGGGAC-3’	331
	R: 5’-GTACTCCAGAAGACCAGAGG-3’	
IL-13	F: 5′-CGCTGGCGGGTTCTGTGTAG-3′	127
	R:5′-GAGGCTGGAGACCGTAGTGGG-3′	
TNF-α	F: 5′-CATCTTCTCAAAATTCGAGTGACAA-3′	175
	R: 5′-TGGGAGTAGACAAGGTACAACCC-3′	
IL-12a (p35)	F: 5′-CCACCCTTGCCCTCCTAAAC-3′	132
	R: 5′-GTTTTTCTCTGGCCGTCTTCA-3′	
IL-12b (p40)	F: 5′-GGAAGCACGGCAGCAGAATA-3′	180
	R: 5′-AACTTGAGGGAGAAGTAGGAATGG-3′	
IL-17A	F: 5′-CAGGGAGAGCTTCATCTGTGT-3′	94
	R: 5′-GCTGAGCTTTGAGGGATGAT-3′	
IFN-γ	F: 5′-AACGCTACACACTGCATCTTGG-3′	237
	R: 5′-GACTTCAAAGAGTCTGAGG-3′	
IL-10	F: 5′-CGGGAAGACAATAACTG-3′	186
	R: 5′-CATTTCCGATAAGGCTTGG-3′	
TGF-β	F: 5′-CAAGGGCTACCATGCCAACT-3′	84
	R: 5′-AGGGCAAGGACCTTGCTG-3′	
IL-35 (ebi3)	F: 5’-CAATGCCATGCTTCTCGGTAT-3’	84
	R: 5’-GGACGTGGATCTGGTGGAGTT-3’	
β-actin	F: 5′-GGCCAACCGTGAAAAGATGA-3′	251
	R: 5′-CACGCTCGGTCAGGATCTTC-3′	

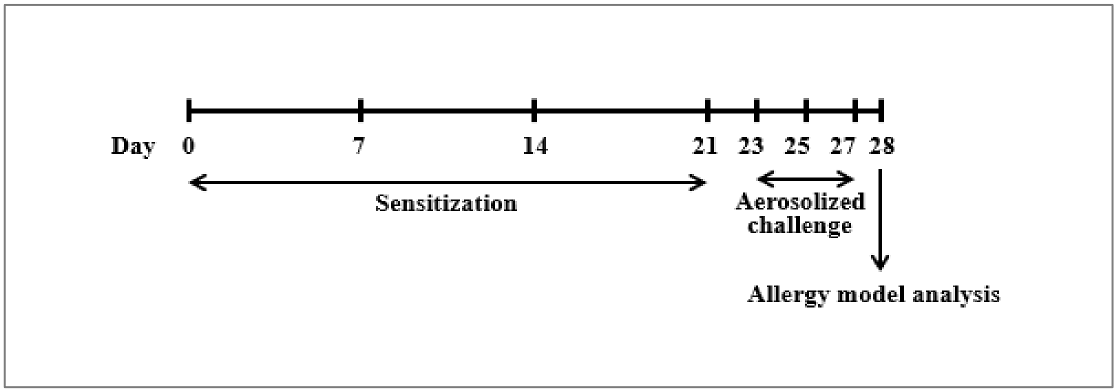

Supplementary Figure 1. Experimental timeline for preparation of allergenized and sham mice.

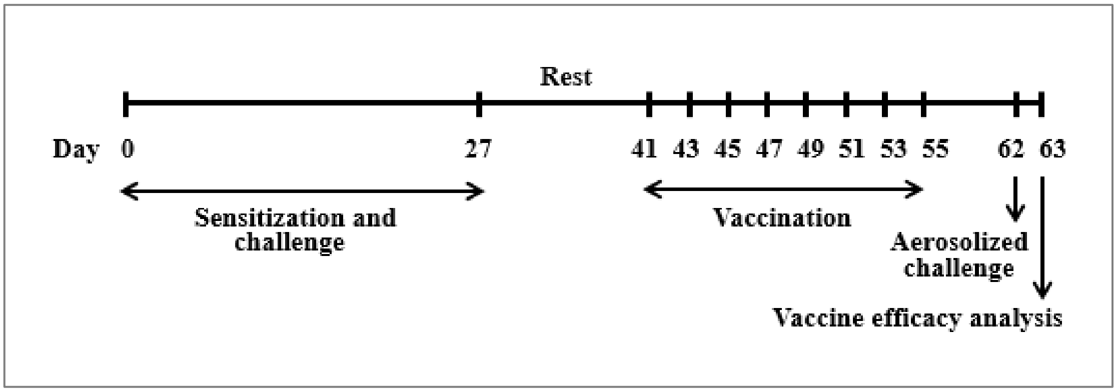

Supplementary Figure 2. Experimental timeline of vaccination (immunotherapy) of allergic mice, allergen provocation (aerosolized challenge) and vaccine efficacy analysis (determination of the therapeutic efficacies of vaccines after vaccination and CRE-provocation).

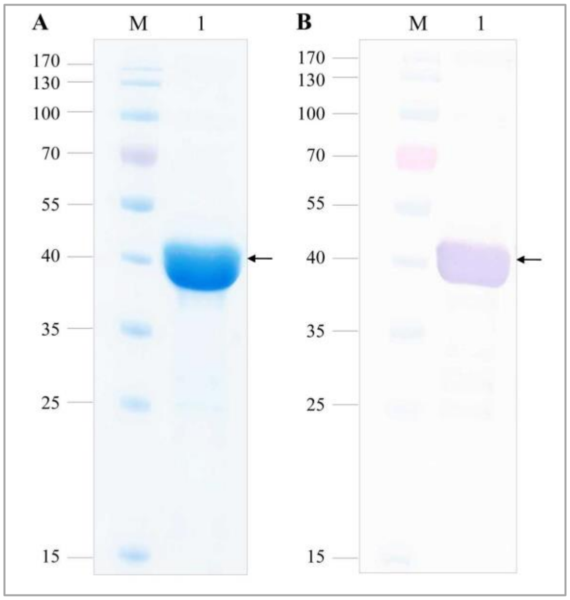

Supplementary Figure 3. Recombinant 6×-His tagged-Per a 9. (A) SDS-PAGE-separated purified recombinant 6×-His tagged Per a 9 (38-40 kDa). (B) Western blot pattern of the SDS-PAGE-separated purified recombinant 6×-His tagged-Per a 9 after probing with anti-6x-His antibody. Lanes M, Protein markers; lanes 1, purified recombinant 6×-His tagged Per a 9. Numbers at the left of (A) and (B) are protein masses in kDa.

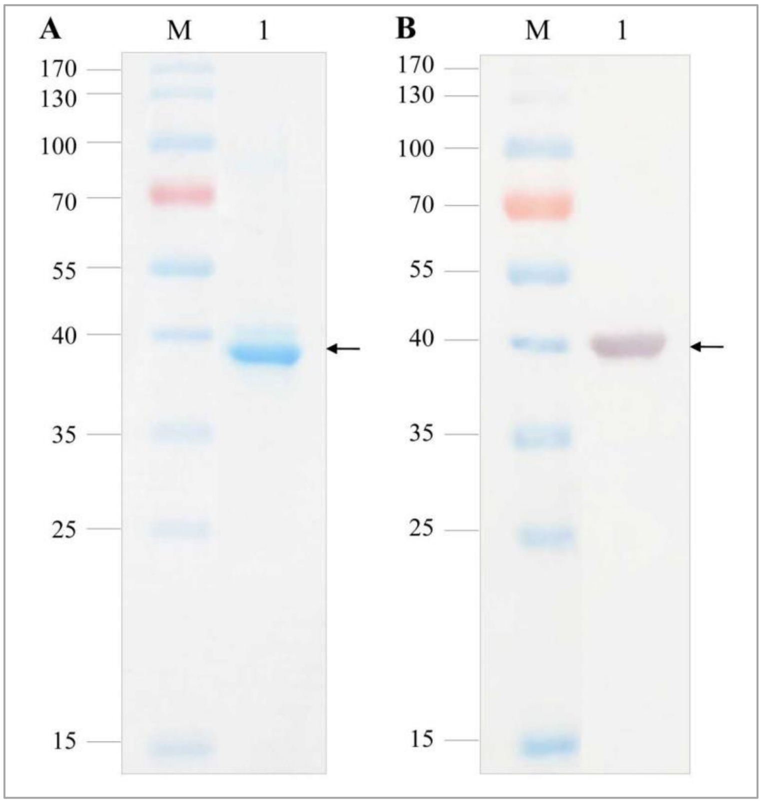

Supplementary Figure 4. Recombinant TGH. (A) SDS-PAGE-separated pattern of recombinant TGH (~38 kDa) after staining with CBB (lane 1); (B) Western blot pattern of SDS-PAGE-separated recombinant TGH probed with anti-6× His antibody (lane 1). Lanes M of (A) and (B), Protein markers. Numbers at the left of (A) and (B) are protein molecular masses in kDa.
